# Case Report: VV-ECMO as a bridge to recovery from ACE inhibitor induced post-obstructive negative pressure pulmonary edema

**DOI:** 10.3389/fmed.2025.1483405

**Published:** 2025-04-29

**Authors:** Darja Smirnova, Eva Steina, Mara Klibus, Edgars Prozorovskis, Eva Strike, Olegs Sabelnikovs

**Affiliations:** ^1^Department of Anesthesiology and Intensive Care, Pauls Stradiņš Clinical University Hospital, Riga, Latvia; ^2^Department of Anesthesiology, Intensive Care and Clinical Simulations, Riga Stradiņš University, Riga, Latvia; ^3^Extracorporeal Organ Support Center, Pauls Stradiņš Clinical University Hospital, Riga, Latvia; ^4^Department of Cardiac Surgery Anesthesia and Intensive Care, Pauls Stradiņš Clinical University Hospital, Riga, Latvia

**Keywords:** case report, angiotensin-converting enzyme inhibitors, angioedema, negative pressure pulmonary edema, venovenous extracorporeal membrane oxygenation

## Abstract

The indications for extracorporeal membrane oxygenation (ECMO) are becoming increasingly widespread nowadays. This case report describes the unique presentation of an adult patient with a combination of two rare complications: life-threatening angioedema caused by angiotensin-converting enzyme inhibitors (ACEi) and subsequent post-obstructive negative pressure pulmonary edema (NPPE). In this case, worsening angioedema that was unresponsive to medication led to severe airway obstruction and near-fatal acute respiratory syndrome due to NPPE. The worsening clinical course required a multidisciplinary approach and immediate initiation of extracorporeal membrane oxygenation (VV-ECMO). The literature reports that most NPPE cases resolve with oxygenation. However, in our case, the NPPE was refractory to mechanical ventilatory support, and we had to initiate VV-ECMO to prevent the patient from going into cardiac arrest due to severe hypoxia. This case underscores the critical role of VV-ECMO as a bridge to recovery from severe NPPE. It also highlights the need to raise clinicians’ awareness of the potential life-threatening side effects of the commonly used antihypertensive drug perindopril.

## Introduction

ACE inhibitors (ACEi) are the first-line treatment for hypertension in adults according to World Health Organization (WHO) recommendations (1) and are among the most commonly prescribed antihypertensive drugs in general practice (2). Although rare (between 0.1 and 0.7% of patients taking the drug) (3), the occurrence of angioedema is a severe side effect caused by a reduction in bradykinin degradation following ACEi administration (4, 5). Mild cases can be successfully treated with the oxygen supply, severe cases usually require short term invasive mechanical ventilation (6, 7).

Negative pressure pulmonary edema or post-obstructive non-cardiac pulmonary edema is a rare complication with an estimated incidence rate of between 0.1 and 12% (8). It usually occurs after a sudden, severe obstruction of the upper airway, e.g., asphyxia, laryngospasm after extubation or endotracheal tube obstruction (8–10). Although ACEi-induced angioedema could potentially trigger NPPE, no documented cases have been reported in the literature to date. In most cases, NPPE resolves with positive pressure ventilation and adequate diuresis. In severe cases, acute respiratory distress syndrome (ARDS) may occur, necessitating invasive mechanical ventilation (8). To date, however, only a few cases requiring extracorporeal membrane oxygenation (ECMO) have been reported (11–13). In the case reports discussed, laryngospasm was the primary trigger for NPPE — occurring post-extubation in two cases [Augustin et al. (11) and Matsumura et al. (12)] and following direct laryngoscopy in a patient with laryngeal papillomatosis [Grant et al. (13)]. According to the ELSO International Registry Report 2022 (14), the use of extracorporeal membrane oxygenation has steadily increased, with indications expanding over time. Considering that NPPE can lead to prolonged and severe ARDS, the application of ECMO in severe, near-fatal NPPE cases is both reasonable and expected.

In this case report, we aimed to present a unique case of severe NPPE following ACE inhibitor use, which was successfully treated with veno-venous ECMO. This case highlights the need for early recognition, prompt airway management and the use of advanced supportive therapies in managing such critical complications.

### Case description

A 72-year-old obese male (173 cm; 110 kg; body mass index, BMI 36.8) presented to the emergency department of Pauls Stradiņš Clinical University Hospital in early Saturday morning. He had a two-hour history of sore throat, bilateral swollen tongue, dysphonia and difficulty swallowing after waking up from sleep due to these complaints. Notably, he had no associated skin rashes or pruritus. Inspiratory stridor, wheezing and rhonchi were absent too. He denied any invasive procedure in the last few months that could be a trigger for angioedema, and there are no known similar cases in his family history.

The patient had a 10-year history of hypertension and diabetes mellitus, managed with daily antihypertensive medication (perindopril for 2 years without recent changes) and metformin. Over the past 18 months, he had experienced three similar episodes of tongue and lip swelling. Two resolved with an antihistamine (*Chloropyramini hydrocloridum*) taken perorally at home, while one required oxygen supply and short-term admission to the secondary health care hospital’s emergency department. All previous episodes were attributed to food allergies and the patient was discharged with the recommendation to consult an allergist and eliminate the allergic product from his daily diet.

He received oxygen therapy via a face mask at 4 L/min, along with glucocorticoids (dexamethasone 12 mg) and intramuscular epinephrine as initial treatment prior to hospitalization. However, these interventions did not lead to clinical improvement and the patient was transferred to hospital. On admission to the emergency department, his vital signs were stable, and all laboratory parameters revealed unremarkable findings. His laboratory analyzes showed normal values for C4, C1 inhibitor protein (C1-INH protein) and C1-INH functions. Given the history of recurrent episodes of angioedema, the absence of laboratory findings suggestive of hereditary angioedema, and the temporal association with ACE inhibitor use, the clinical diagnosis of perindopril-induced angioedema was made.

Over the next 6 h in the emergency department, the patient’s respiratory status deteriorated and oxygen therapy was increased to a high-flow mask at 10 L/min. Additional treatment with high-dose glucocorticoids (Solumedrol 500 mg), antihistamines (*Chloropyramini hydrochloridum* 20 mg), intravenous epinephrine, tranexamic acid (1 g), and two units of fresh frozen plasma was administered as part of the management strategy for severe ACEi-induced angioedema. However, these interventions did not lead to any improvement. Secondary physical examination revealed marked swelling of the tongue and increasing shortness of breath. As the angioedema continued to worsen despite medical treatment, the decision was made to proceed with invasive mechanical ventilation. However, due to difficult intubation, an acute tracheostomy was performed. The patient was then transferred to the intensive care unit (ICU) for further management and mechanical ventilation. Despite these interventions, he remained dependent on a high oxygen concentration to maintain adequate oxygenation, requiring an FiO2 of 0.7.

On the second day, despite maximum ventilatory support, the patient developed progressive hypoxia. On physical examination, his face and neck were cyanotic, and oxygen saturation was 80–82% with a 100% fraction of inspired oxygen (FiO2). The patient was agitated, and to optimize patient-ventilator synchronization, continuous infusions of fentanyl, midazolam, propofol, and cisatracurium were initiated. Optimal diuresis (>1.1 mL/kg/h) achieved with 80 mg furosemide and lung protective ventilation was initiated without any improvement in respiratory status. Despite maximal medical optimization, hypoxia continues to worsen with P/F ratio values ranging between 39 and 43 and SpO2 from 63 to 72% at 100% FiO2 over the next 6 h.

**Figure 1:**
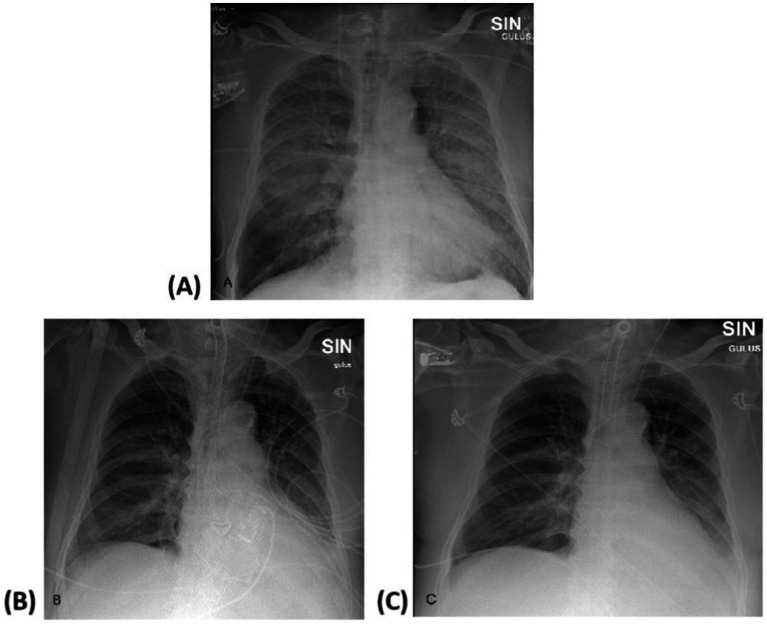
Portable chest X-rays. **(A)** X-ray taken on ICU day 2 (prior VV-ECMO cannulation). This radiograph demonstrates acute lung failure, with almost complete opacification of both lung fields. The bronchovascular pattern is accentuated, with uneven alveolar infiltrative changes bilaterally, more pronounced on the left side, suggesting pulmonary edema. The lung roots are homogenized, and the diaphragmatic domes are visible. There is no significant volume of contents in the pleural spaces. The heart and aorta shadows appear of normal size and configuration. **(B)** Day 8, before decannulation of VV-ECMO: The ECMO cannula is visible in the right internal jugular vein, with its tip reaching the distal third of the superior vena cava. Slight bronchovascular infiltrative changes are seen in the perihilar regions. Bilateral pulmonary ventilation has further improved, and the volume and intensity of atypical infiltrative changes have further decreased. **(C)** Day 9, after VV-ECMO decannulation: Compared to the previous examination, there is a significant improvement in bilateral lung ventilation, with a decrease in both the volume and intensity of atypical infiltrative changes.

The portable chest X-ray showed bilateral lung field opacification (Figure 1A). The level of B-type natriuretic peptide (BNP) was 112 pg./mL. Transthoracic echocardiography was unremarkable, showing only a slightly enlarged left atrium with a preserved ejection fraction of 55%, making cardiogenic pulmonary edema an unlikely cause of respiratory deterioration. A computed tomography scan of the chest performed on admission to the intensive care unit revealed no evidence of aspiration, secondary pulmonary infection or associated complications. The patient showed no signs of inflammation, as inflammatory markers were not elevated (C-reactive protein 23.59 mg/L, interleukin-6 0 pg./mL, procalcitonin 0.13 ng/mL) and microbiologic cultures were sterile. In addition, bronchoscopy revealed the presence of pink foamy fluid. These findings, in the context of difficult airway management, strongly suggested NPPE as the primary contributor to the development of ARDS.

**Figure 2:**
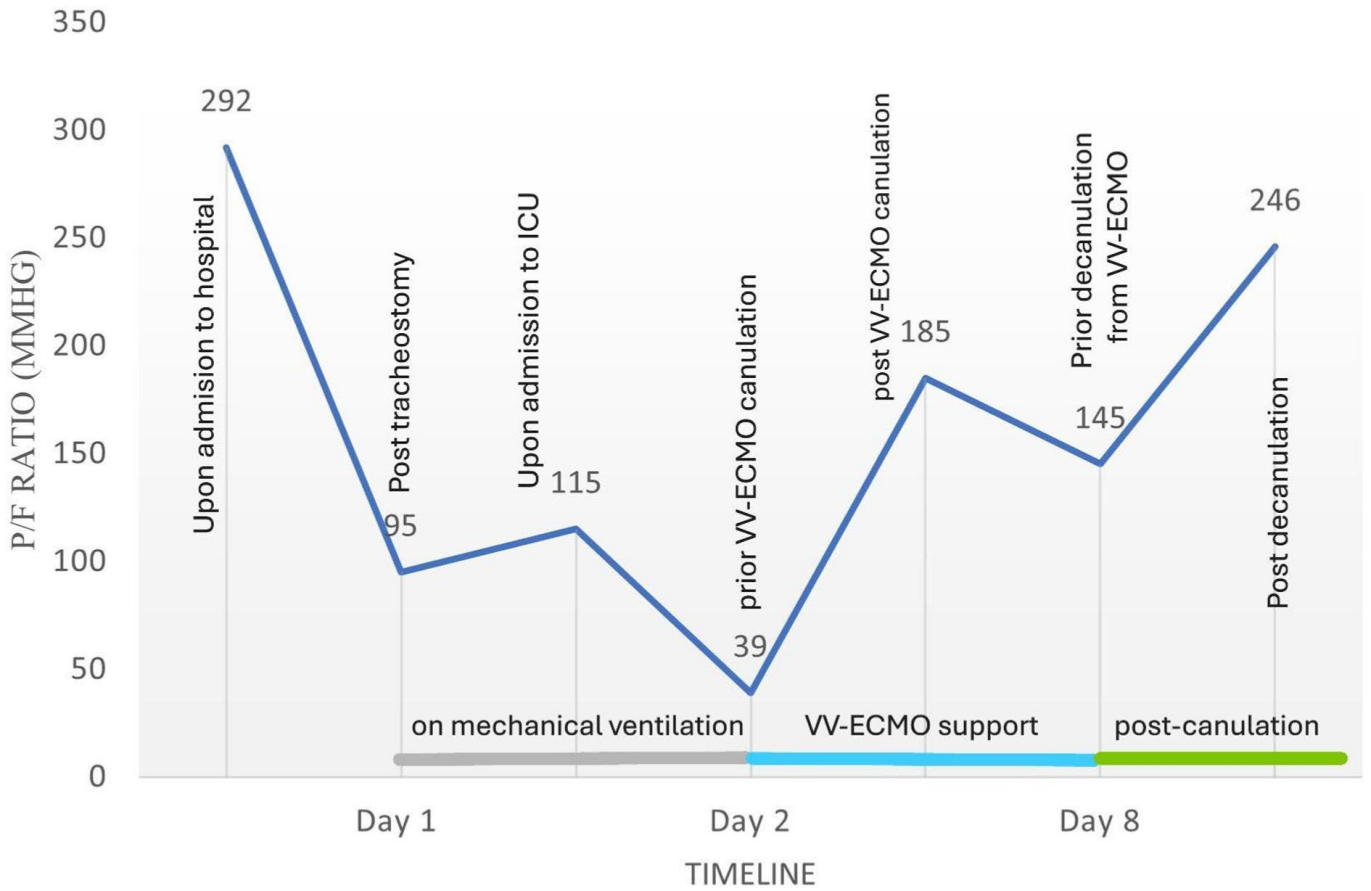
Flow chart illustrating the course of the PaO₂/FiO₂ (P/F) ratio in relation to key clinical events.

Despite the treatment applied, including optimal diuresis, synchronization with mechanical ventilation and maximal oxygen support, the patient remained in severe hypoxia (P/F ratio as low as 39 at FiO2 100%), leading to hemodynamic instability and bradycardic episodes. For this reason, the ECMO team specialists were consulted. The decision to initiate veno-venous ECMO for respiratory support was made based on the patient’s Respiratory Survival Prediction Score (RESP) of zero, risk class III and a predicted in-hospital survival rate of 57%. Due to the patient’s constitution, the right femoral vein was cannulated with a 29 Fr cannula for venous access and the right internal jugular vein was cannulated with a 21 Fr cannula for venous return, followed by a heparin bolus of 10,000 units. ECMO was initiated via MAQUET Cardiohelp System. As the patient was hypoxic and normocapnic, the initial ECMO pump flow was 3 L/min at 2200 RPMS and the «sweep gas» was 3 L/min with a FiO2 of 100%. The ventilator settings were adjusted to lung-protective ventilation (FiO2 40%, PEEP 10 cm H2O, RR 10, tidal volume (Tv) 450 mL) throughout the ECMO period. Immediately after ECMO support was initiated, serial arterial blood gas (ABG) analyzes showed a rapid improvement in the patient’s oxygenation (Table 1, Figure 2) and vital signs. To maintain ECMO support, heparin anticoagulation was administered, targeting an aPTT goal of 40–60 s. The circuit was set to a flow of 3.5–4.0 L/min with a FiO2 of 100%, RPMs of 2,200–2,400, and a sweep gas flow of 3–4 L/min, adjusted according to the ABG analysis.

**Table 1:** Dynamics of arterial blood gas analysis during major clinical events.

	pH	pO2 (mmHg)	pCO2 (mmHg)	Fi02	P/F ratio (mmHg)	Sp02 (%)
On admission to emergency department (Day 1)	7.32	117	36	0.4	292	100
Prior to mechanical ventilation (Day 1)	7.18	62	56	0.65	95	84
In the intensive care unit (Day 1)	7.33	81	36	0.7	115	97
Prior to VV-ECMO cannulation (Day 2)	7.39	39	36	1.00	39	48
Immediately post VV-ECMO cannulation (Day 2)	7.35	74	39	0.4	185	95
Prior de-cannulation (Day 8)	7.46	87	36	0.6	145	97
Post de-cannulation (Day 8)	7.42	148	39	0.6	246	99

The FiO₂ values shown in the table are taken from the settings of the invasive mechanical ventilators. The bold values presented in Table 1 highlight key clinical turning points, including marked deterioration prior to mechanical ventilation, severe hypoxemia before VV-ECMO cannulation, and a subsequent rapid improvement in respiratory parameters during and after VV-ECMO therapy—supporting its role as an effective bridge to recovery.

Over the next 6 days, the chest x-ray and hypoxia (Figures 1B,C) gradually improved and VV-ECMO was discontinued on day eight after admission to the hospital. He was further weaned from mechanical ventilation over the next 10 days and transferred to the ward on day 18 without the need for oxygen therapy. He was discharged home on day 23 after rehabilitation (Table 2) with a recommendation to consult a geneticist to screen for known mutations underlying a possible HAE-nC1-INH. Three months after discharge from our hospital, the patient stated in a telephone interview that he had resumed his daily life without sequelae. However, the patient had not yet undergone genetic counseling by the time of the follow-up examination, which was justified by a lack of necessity. After discharge, treatment with the angiotensin II receptor blocker telmisartan was initiated, with the dose being gradually increased to 80 mg once daily. No allergic reactions were observed in the following 3 months and hypertension remained well controlled.

**Table 2:** Timeline of relevant clinical events.

Day	Events
1	Admission to the emergency department (early morning) Treatment: Methylprednisolone, Epinephrine, antihistamines, fresh frozen plasma transfusion, tranexamic acid. Oxygen supply with low concentration face mask
	Worsening angioedema unresponsive to medication Acute tracheostomy, transfer to the intensive care unit (early afternoon). Lung protective mechanical ventilation (tidal volume of 6 mL/kg, a respiratory rate of 10 to 16 breaths per minute, a positive end-expiratory pressure (PEEP) of 8 cmH2O and a plateau pressure of 26 cmH2O)
2	X-ray with opacification of the bilateral lung fields Sedation, loop diuretics for optimal diuresis VV ECMO cannulation (late afternoon, 42 h after admission to hospital)
3–8	Improvement of the respiratory status Decannulation of VV-ECMO on day 8
18	Oxygen therapy no longer required Transfer to the pulmonary department
23	Discharge to home

An overview of the main clinical events is provided, with the table also listing the main interventions during the course of admission to the intensive care unit and throughout hospitalization.

On the first day, there was a small amount of bleeding from the cannulation site of the venous ECMO access, but this was resolved by suturing and did not require transfusion of blood components. No further complications related to ECMO occurred during the treatment period.

## Discussion

The clinical course of the presented case is highly unusual and includes two rare but severe complications: angioedema induced by an ACE inhibitor perindopril and subsequent post-obstructive negative pressure pulmonary edema, successfully managed with VV-ECMO.

Angiotensin-converting enzyme inhibitors, such as perindopril, are widely prescribed for managing hypertension and other cardiovascular diseases, yet they can cause severe side effects (4, 5). Although angioedema is a rare side effect of ACE inhibitors and affects only a small percentage of patients (3), its clinical significance remains high due to the widespread use of ACEi as a commonly prescribed medication.

The pathophysiology of the development of all forms of angioedema is complex and can be defined as either hereditary (bradykinin-mediated) or acquired (histamine-or bradykinin-mediated angioedema). ACEi-induced angioedema is an acquired bradykinin-mediated angioedema and is associated with an accumulation of bradykinin (4, 5). As a vasoactive peptide, bradykinin increases vasodilation and alters vascular permeability, which can lead to life-threatening airway obstruction, as seen in our patient. Typical laboratory findings of ACEi-induced angioedema include normal levels of C1-INH function, C1-INH protein and C4, as in our case. This laboratory finding helps to differentiate acquired bradykinin-mediated angioedema from hereditary angioedema (HEA) type I or type II, which is caused by a mutation in the SERPING1 gene encoding C1-INH and therefore will presented with low levels of C1-INH and C4 (15, 16). However, it does not help to exclude the very rare disease as HEA type III or HEA with normal values of C1-INH (HAE-nC1-INH) (16–18). Therefore, the WAO/EAACI international guideline for the management of hereditary angioedema recommends that patients suspected of having HAE who have normal C1-INH levels and function should be screened for known mutations underlying HAE-nC1-INH (15).

Distinguishing between different forms of angioedema is always challenging but crucial as the underlying pathophysiology influences clinical presentation and treatment recommendations. Bradykinin-mediated angioedema tends to be more severe, longer lasting and often results in upper airway swelling without urticaria, compared to histamine-mediated or allergic angioedema (7, 19, 20). According to the literature, the most important part of the treatment protocol for ACEi angioedema involves immediate cessation of the trigger (in our case perindopril) (4–6, 21). However, antihistamines, corticosteroids, and epinephrine are also prescribed to counteract vasodilation and increased vascular permeability (19). Although these medications do not directly affect bradykinin levels, they are commonly administered for ACEi-induced angioedema. This practice can be attributed to the challenges in establishing a definitive diagnosis and the urgency of initiating treatment. From a clinical practice perspective, the absence of a specific laboratory marker for the rapid diagnosis of angioedema necessitates a reliance on clinical factors. A lack of response to antihistamines, a history of ACE inhibitor use and the rapid onset of upper airway swelling may be helpful in identifying ACEi-induced angioedema (21).

In recent years, several new drugs have been developed for the treatment of hereditary angioedema. Some have also been investigated for ACEi-induced angioedema, based on the suspected common pathophysiological mechanism — excessive accumulation of bradykinin. Although specific therapy with Icatibant, a bradykinin B2 receptor antagonist currently approved for the treatment of HAE (15), has shown inconsistent results in the treatment of ACEi-induced angioedema, as evidenced by a recent meta-analysis of randomized controlled trials (20, 22). In some cases, an additional treatment strategy - tranexamic acid, fresh-frozen plasma transfusions and C1-esterase inhibitor concentrate - has shown beneficial results, also well-designed trials are lacking (18, 23–25).

The administration of Icatibant or C1-esterase inhibitor concentrate was not feasible in the clinical case we reported. The event occurred in the early morning hours of a weekend when the medication was not available in the emergency department. Current evidence suggests that Icatibant is most effective when administered immediately, as delayed administration reduces its efficacy (26–28). In addition, there is no high-quality randomized trial confirming the efficacy of Icatibant, and due to its high cost, this therapy is not included in the local treatment protocol for ACEi-induced angioedema. These factors further limited the options for specific treatment as the condition progressed.

In the reported case, the angioedema was refractory to treatment and led to severe airway obstruction requiring emergency tracheostomy. The patient’s condition continued to deteriorate, leading to the development of severe ARDS with a near-fatal P/F ratio of 39, indicating critical hypoxemia and extreme respiratory failure.

We identified negative pressure pulmonary edema (NPPE) as the main cause of ARDS. Negative pressure pulmonary edema is a complication resulting from acute or chronic upper airway obstruction and often presents challenges to clinicians in recognition and diagnosis. NPPE occurs when significant upper airway obstruction generates highly negative intrathoracic pressure leading to pulmonary edema. The clinical manifestations of negative pressure pulmonary edema include dyspnea, tachypnea, cyanosis, and the production of a profuse pink foamy sputum in combination with hypoxia and hypercapnia as a result of underlying respiratory dysfunction and impaired gas exchange. All these clinical features were evident in our case.

However, consideration of possible differential diagnoses is crucial for appropriate management. One of the differential diagnoses is cardiogenic pulmonary edema, which can present with similar symptoms (29). In our case, however, the echocardiographic examination revealed no evidence of systolic or diastolic dysfunction of the left ventricle, no significant valvular abnormalities and normal left atrial pressure. In addition, the level of BNP was slightly increased. Based on these findings, a cardiogenic etiology was effectively ruled out. The second differential diagnosis is infectious pneumonia, including aspiration pneumonia, which is a relevant consideration given the patient’s difficult airway. Although aspiration was a potential concern, the absence of characteristic CT findings, normal inflammatory markers, and sterile respiratory cultures made an infectious process unlikely (30). Another potential differential diagnosis in our case is aspiration pneumonitis, especially considering the difficult intubation. In contrast to aspiration pneumonia, aspiration pneumonitis is caused by sterile gastric contents causing chemical lung injury. Clinically, it presents with acute hypoxemia, tachycardia, fever and bilateral infiltrates, often following an aspiration event, typically in unconscious patients (30, 31). In our case, no massive aspiration was detected during bronchoscopy after intubation. However, since even minimal gastric content can cause lung injury and the clinical and radiologic features overlap with NPPE, we cannot entirely exclude aspiration pneumonitis as a contributory cause for the development of severe ARDS. Nevertheless, given the clinical course and supportive diagnostic findings, NPPE remained the most plausible etiology of ARDS in our clinical case.

Although most NPPE cases respond well to conservative treatments like oxygen therapy and mechanical ventilation (9), our patient’s NPPE was refractory to mechanical ventilation, leading to severe hypoxemia and the imminent risk of cardiac arrest associated with near fatal bradycardia. This rare and severe presentation necessitated the initiation of veno-venous ECMO. Only some cases of severe NPPE were managed with ECMO, according to the previous literature (11–13). However, none of them were caused by ACEi angioedema. In this case, VV-ECMO provided the essential respiratory support needed to maintain adequate oxygenation and allowed the lungs to rest and recover, demonstrating its critical role in managing severe respiratory failure unresponsive to conventional treatments.

The management of this case required a multidisciplinary approach with the involvement of intensivists and ECMO specialists. Both the timely recognition of the severity of the clinical course and the decision to initiate VV-ECMO were crucial for the stabilization of the patient. This case demonstrates the importance of a well-coordinated ECMO team within the hospital that is able to rapidly deploy this life-saving technology. The expertise and preparedness of such a team can significantly improve outcomes for patients with severe, refractory respiratory failure.

Effective data transmission and availability among all healthcare providers involved in a patient’s care is critical to the management of severe clinical conditions. In this case, the patient had previously experienced similar symptoms three times, which recurred 6 months after initiating perindopril. However, due to inadequate communication, this information did not reach the patient’s primary care physician and ACEi was not discontinued. Strategies aimed at improving physician and patient education regarding ACE inhibitor cessation after primary angioedema, along with proper documentation of adverse drug reactions in medical records are essential. These measures can help prevent severe complications and reduce the need for critical interventions such as VV-ECMO.

The management of hypertension in patients with a history of ACEi induced angioedema requires careful consideration. The main step is to discontinue the ACEi and switch to an alternative agent. Angiotensin II receptor blockers (ARBs) are often recommended due to their significantly lower risk of angioedema compared to ACEi. However, ARBs should be started at a low dose and titrated gradually under close monitoring, as cross-reactivity between ACEi and ARBs is possible. If ARBs are not appropriate or there is a high risk of recurrence, other antihypertensive options such as calcium channel blockers or thiazide diuretics should be considered (3, 32).

In summary, this case highlights the importance of early recognition and aggressive management of ACEi-induced angioedema and underscores the potential of ACE inhibitors to cause life-threatening complications. Increased awareness of the severe side effects of commonly prescribed medications such as perindopril can lead to better patient outcomes through prompt and appropriate intervention. In addition, awareness of NPPE as a potential consequence of severe airway obstruction is critical. While NPPE typically responds to conservative measures, this case demonstrates that refractory cases may require advanced interventions such as VV-ECMO.

## Conclusion

We present the case of an obese middle-aged man with a unique clinical course of negative pressure pulmonary edema due to severe upper airway obstruction caused by perindopril angioedema. This case highlights the need for data sharing between healthcare providers involved in the patient’s treatment to improve early detection of potential complications and qualitative management. Raising awareness of the potential side effects of commonly prescribed medications such as perindopril and the potential complications of angioedema can improve patient outcomes through early recognition and appropriate intervention. This case also emphasizes the benefits of timely initiation of veno-venous ECMO for respiratory support in severe refractory NPPE cases such as this one. This broadens the indications for extracorporeal membrane oxygenation and emphasizes the need for a well-coordinated ECMO team within the hospital.

## Data Availability

The original contributions presented in the study are included in the article/supplementary material, further inquiries can be directed to the corresponding author.
